# Does having more power make people more materialistic? The role of personal sense of power for gift preferences

**DOI:** 10.3389/fpsyg.2023.1235527

**Published:** 2023-08-25

**Authors:** Shichang Liang, Xiaoyan Han, Xueying Yuan, Meiting Liang, Yiwei Zhang, Zhen Liu, Pin Xie

**Affiliations:** School of Business, Guangxi University, Nanning, China

**Keywords:** personal sense of power, gift, material gift, experiential gift, information processing fluency

## Abstract

**Introduction:**

Gift-giving is a prevalent practice in daily life, with experiential gifts being identified in studies as having hedonic and interpersonal advantages, often yielding greater recipient satisfaction compared to material gifts. However, the reception of experiential gifts might not always align with expectations, as material gifts are valued for their enduring qualities. Thus, comprehending the contexts favoring material or experiential gift preferences becomes crucial.

**Methods:**

Existing research primarily delves into external influences like income and social proximity, while intrinsic factors such as personal sense of power in interpersonal interactions have received limited attention. Guided by the Agentic-communal Model of Power, we conducted three studies to investigate how personal sense of power impact gift preferences.

**Results:**

Our findings demonstrated that gift preferences are contingent upon personal sense of power. Specifically, those possessing a high personal sense of power exhibited a preference for material gifts over experiential ones, whereas individuals with a low personal sense of power favored experiential gifts over material ones. Further analysis revealed that the relationship between personal sense of power and gift preference is mediated by information processing fluency.

**Discussion:**

This study contributes to the field of gift preferences and sheds light on the role of personal sense of power. By incorporating the Agentic-communal Model of Power, we offer novel insights into the dynamics between personal sense of power and gift preferences. These findings hold valuable implications for managerial strategies concerning gift selection and interpersonal interactions.

## Introduction

Every year, numerous occasions call for gift-giving, such as traditional holidays, birthdays of friends and family, and more (Belk and Coon, 1993). The act of exchanging gifts serves to establish and strengthen bonds with loved ones (Galak et al., 2016; Goodman et al., 2016). Gifts are not merely a means for the giver to convey information, express emotions, and extend well-wishes to the recipient, they also bring joy and happiness to the receiver (Pilwha, 2016). Studies have shown that giving a gift can significantly increase the recipient’s sense of happiness and enjoyment (Belk and Coon, 1993). Unfortunately, gifts sometimes do not receive the expected reception from the recipient (Adams et al., 2012). Choosing the wrong gift can harm a relationship (Roster, 2006; Dunn et al., 2008). Therefore, it’s essential to consider the recipient’s preferences when selecting a gift. Meanwhile, Individuals often encounter varying degrees of personal sense of power in life situations. Research in consumer behavior has demonstrated that a personal sense of power is critical to consumer decision-making (Guinote, 2007). Therefore, examining whether the personal sense of power influences gift preferences is important. This paper aims to address this questions.

Gift preferences have been a topic of great interest to researchers for a long time (Waldfogel, 1993; Teigen et al., 2005; Flynn and Adams, 2009; Gino and Flynn, 2011; Zhang and Epley, 2012). Research has shown that gift recipients are only delighted when the gift aligns with their personal preferences (Gino and Flynn, 2011). The current literature on gift preferences focuses on factors such as gender (Parsons, 2002; Pollmann and van Beest, 2013), age and income (Parsons, 2002), interpersonal orientation (De Hooge, 2017), and culture (Wang, 2007; Aung et al., 2017). However, insufficient emphasis has been placed on the personal sense of power regarding gift preferences. Recently, there has been a considerable focus on the notion of a personal sense of power, which is now recognized as a pervasive and influential element in social dynamics, ultimately impacting interpersonal connections (Fiske, 1993; Guinote, 2007; Feng, 2011). Researchers have started to recognize the psychological aspects of power, conceptualizing it as a subjective psychological experience known as the personal sense of power (Anderson and Berdahl, 2002; Anderson et al., 2012; Sijbom and Parker, 2020). Studies have revealed that individual differences in the personal sense of power can affect various aspects, such as perceptions, cognition, consumer behavior, and purchase intentions (Guinote, 2007). For instance, an individual’s personal sense of power can shape their product preferences. Those with a low personal sense of power may favor products that symbolize high status, such as luxury branded cars, designer watches, and silk ties (Rucker and Galinsky, 2008). They may also prefer products with a wide range of options (Inesi et al., 2011) and larger-sized products (Dubois et al., 2012). However, the impact of the personal sense of power on gift preferences has received less attention from scholars. This paper investigates whether the personal sense of power affects gift preferences and explores the underlying mechanisms. Doing so will address the gap in the existing literature regarding the relationship between a personal sense of power and gift preference.

Many researchers view the act of receiving gifts as a form of consumer behavior, and they often draw on marketing relationship theories and concepts to explain the underlying process involved in gift consumption (Davies et al., 2010). Previous scholars have utilized Van Boven and Gilovich’s conceptualization of material and experiential purchases (Van Boven and Gilovich, 2003) to classify gifts into material and experiential. Material gifts are defined as preservable, preservable physical items, such as books, jewelry, and clothing. On the other hand, experiential gifts refer to non-preservable experiences or activities provided to individuals, such as travel, concert tickets, and cooking classes (Goodman and Lim, 2018). According to Goodman’s research results (Goodman et al., 2016), Studies have shown that experiential gifts can boost consumer well-being. Furthermore, experiential gifts possess uniqueness and reflect the giver’s personality that are conveying concern and sending warm signals (Belk and Coon, 1993). As a result, experiential gifts may have a more significant emotional impact on the recipient than material gifts. Material possessions are often closely associated with money, while material gifts convey benefit values more closely than experiential gifts (Kumar and Gilovich, 2015).

Meanwhile, according to Rucker et al. (2012), the Agentic-Communal Model of Power posits that individuals with a high personal sense of power are likelier to exhibit an agentic orientation. This orientation can lead them to become more self-focused, concerned with their feelings (Rucker et al., 2011), and attentive to material possessions that reflect their interests and values (Rucker et al., 2011). Therefore, this article proposes that individuals who experience a high personal sense of power tend to place greater importance on material gifts that offer practical benefits. Conversely, those with a low personal sense of power typically prioritize their relationships with others and adopt a more communal mindset. Consequently, individuals may assign more significance to the present’s social and emotional worth (Rucker et al., 2011). Moreover, a communal mindset can lead individuals to prioritize the warmth conveyed in a message (Dubois, 2016). Thus, this paper proposes that individuals with a low personal sense of power exhibit a greater inclination toward experiential gifts that effectively convey a profound emotional impact from the giver. In addition, according to the research of Aaker (Cesario et al., 2004; Aaker and Lee, 2006), information processing fluency mediates the relationship between a personal sense of power and gift preference.

This study makes three critical theoretical contributions. First, this article expands the research on a personal sense of power to the gift preference. Prior research examined the influencing factors of gift preference mainly focused on extrinsic factors such as income and social distance, with less attention given to intrinsic factors such as personal sense of power in interpersonal interactions. This article finds that individuals who experience a high personal sense of power tend to place greater importance on material gifts that offer practical benefits. Second, this article enriches the literature on information processing fluency as a mediating variable, exploring the effect of the personal sense of power on gift preference using information processing fluency as a mediating variable. Finally, our research offers important managerial implications. Our findings guide gift-givers and insights into individuals’ gift preferences (material vs. experiential) as gift recipients.

## Theory and hypotheses

### Material gifts and experiential gifts

A gift is given voluntarily by one person or organization to another, often through a ceremony or formal presentation (Belk and Coon, 1993). Giving gifts serves a purpose beyond economic exchange; it also expresses selfless love and plays a vital role in maintaining interpersonal relationships (Jacobs, 1979). Research has indicated that gifts can communicate economic and functional value and social value, reflecting or influencing the relationship between the giver and the recipient. Therefore, a gift’s social value is essential when evaluating its meaning and significance (Larsen and Watson, 2001; Antón et al., 2014).

Referring to the characterization of material and experiential purchases provided by Van Boven and Gilovich (2003), the researchers categorized gifts into two types: material gifts and experiential gifts (Baskin, 2014). Material gifts pertain to tangible items, such as clothing and watches. Conversely, experiential gifts involve intangible items like visiting an art exhibition or attending a concert. While research on material gifts and experiential gifts has mainly focused on consumers’ purchase feelings, specifically the differences between experiential purchases and material purchases. Previous studies have consistently shown that experiential purchases, rather than the acquisition of material purchases, tend to impact individuals positively (Kok and Fredrickson, 2010). Compared to material purchases, experiential purchases can lead to greater satisfaction (Rosenzweig and Gilovich, 2012)and more pleasure (Goodman and Lim, 2018). In addition, experiential purchases have more social value (Van Boven and Gilovich, 2003) than material purchases (Kumar and Gilovich, 2015). Experiential gifts are typically more unique and personalized (Rosenzweig and Gilovich, 2012), making them less comparable in value to other alternatives (Carter and Gilovich, 2010). By offering an experiential gift, the giver expresses a more profound concern and sends warm signals to the recipient. Furthermore, because recipients tend to experience intense emotions when consuming experiential gifts, the type of gifts is believed to enhance relationships more than material gifts. Whether the giver and recipient share the gift or not, experiential gifts are thought to facilitate further improvements in the relationship (Chan and Mogilner, 2016). Moreover, previous studies have shown that experiential gifts have the potential to enhance the social connection between the giver and the recipient of the gift (Chan and Mogilner, 2016). Giving experiential gifts is also a powerful way to engage in pro-social consumption (Puente Díaz and Cavazos, 2021). Experiential gifts may communicate more emotional meaning to the recipient than material gifts and warmth signals. Because material items can be physically held and kept for a long time, they tend to be more memorable (Goodman and Lim, 2018). They often link to their monetary value (Kumar and Gilovich, 2015). As a result, material gifts may more closely convey a message of benefit than experiential gifts.

Overall, the existing research on experiential and material gifts primarily focuses on identifying the types of gifts that enhance personal well-being and exploring the connections and differences between these two categories of gifts. Although prior studies have highlighted the advantages of experiential and material gifts, there has yet to be a clear consensus on gift preferences in various contexts. Additionally, previous research needs to pay more attention to the personal sense of power. As a result, this paper aims to investigate that gift preferences based on their personal sense of power.

### Personal sense of power

A personal sense of power refers to an individual’s subjective perception of their relative ability in social relationships based on the perceived possession of valuable resources that are asymmetrically distributed (Tiedens, 2001; Keltner et al., 2003; Lin et al., 2019). Individuals often encounter varying degrees of personal sense of power in different life situations. Research in consumer behavior has demonstrated that a personal sense of power is critical in consumer decision-making. The personal sense of power affects consumers’ psychological perceptions (Tiedens, 2001), values (Garbinsky et al., 2014), behavioral intentions (Kim et al., 2017), and information processing and persuasion (Dubois, 2016). The personal sense of power discussed in this paper is a psychological state. It can be impacted by contextual factors, societal roles, and past recollections of prior encounters involving power, ultimately determining whether an individual feels empowered or disempowered (Galinsky et al., 2006). For instance, specific actions such as rating a new employee, directing a subordinate to correct a task, or instructing and providing feedback in a classroom can evoke a high personal sense of power. In contrast, actions like being interviewed and assessed, speaking to a superior, or requesting someone to wait in line for service can lead to a low personal sense of power.

The Agentic-Communal Model of Power is a power framework that explains how personal sense of power influences and directs consumer behavior by emphasizing the simultaneous orientations of agency and communion (Rucker et al., 2012). The model reveals that individuals with high personal sense of power tend to have a heightened sense of control over resources. Consequently, they naturally lean toward an agentic orientation, prioritizing self-expression and self-promotion. Conversely, individuals with low personal sense of power rely on others for valuable resources, leading them to adopt a more communal orientation. As a result, they are more inclined to focus on nurturing relationships and taking others’ feelings into account.

As research on a personal sense of power progresses, it has become increasingly evident that individual variations in this trait significantly impact people’s perceptions, cognitive processes, consumer behavior, and purchase intentions (Guinote, 2007). A review of the literature shows that a personal sense of power influences their preferences for consumer products. Research on compensatory consumption suggests that individuals with a low personal sense of power draw to status goods, which provide symbolic meaning that compensates for the psychological threat of a low personal sense of power. Consequently, these consumers purchase products that symbolize status (Rucker and Galinsky, 2008). Consumers with a high personal sense of power tend to prioritize the functional attributes of products. In contrast, those with a low personal sense of power tend to focus more on conspicuous attributes. Furthermore, according to Dubois, consumers with a low personal sense of power are prefer to purchase more oversized products when size is associated with status symbolism (Dubois et al., 2012). This effect has been observed in the context of compensatory consumption and other consumption behaviors. For instance, people with a high personal sense of power tend to believe they can control others, making them less risk-averse regarding anthropomorphic goods. Consequently, they are more inclined to try buying such products than those with a low personal sense of power (Kim, 2011). Individual differences in the perception of personal power can significantly affect how consumers view price inequity. Specifically, individuals with a higher personal sense of power tend to compare themselves to others more often due to their greater access to resources. As a result, they are more likely to feel a sense of unfairness when they pay more for goods than others (Jin et al., 2014).

In conclusion, previous studies have primarily examined the impact of the personal sense of power on consumers’ product preferences and price perceptions while neglecting to explore the influence of personal sense of power on individual gift preferences (experiential gifts vs. material gifts). As such, this paper aims to expand on the existing research on a personal sense of power by exploring its influence on gift preferences and investigating the mechanisms that underlie this relationship.

### Personal sense of power and gift preferences

Scholars have presented different interpretations and viewpoints of gift preferences. The factors influencing gift preferences can be understood from multiple perspectives (Waldfogel, 1993; Flynn and Adams, 2009; Gino and Flynn, 2011; Zhang and Epley, 2012; Choi et al., 2018; Rombach et al., 2021)，which are divided into two main categories: extrinsic factors and intrinsic factors. Prior research has found no significant association between the monetary value of a gift and the recipient’s level of appreciation, as evidenced by the study conducted by Flynn and Adams (2009). Recipients often value gifts less than they cost (Waldfogel, 1993)，suggesting that a gift’s value is not necessarily tied to its price and that more expensive gifts are not necessarily better preferences. The gift’s value is not just determined by its material worth but also by the message and meaning it conveys, as well as how it reflects certain aspects of the characteristics of both parties involved (Larsen and Watson, 2001; Antón et al., 2014). The gift’s value is an essential factor that contributes to the recipient’s satisfaction with the gift. Moreover, research suggests that individuals prefer receiving cash or gifts they have specifically requested rather than unsolicited presents (Gino and Flynn, 2011). Studies also discovered that social distance influence gift preference, with recipients displaying a stronger inclination toward experiential gifts when the giver is socially closer to them (Goodman and Lim, 2018). Meanwhile, the construal level of the individual can affect the gift preferences. Individuals with a high construal level typically choose gifts with high desirability, such as gifts of high quality. In contrast, recipients with a low construal level tend to favor gifts that are highly feasible and convenient (Baskin, 2014). In addition, studies have found that gift recipients’ age and income (Parsons, 2002), gender (Parsons, 2002; Pollmann and van Beest, 2013), interpersonal orientation (De Hooge, 2017), and culture (Wang, 2007; Aung et al., 2017) can all have an impact on gift preferences.

People with a high personal sense of power typically have access to a greater abundance of tangible and intangible resources (Keltner et al., 2003; Scholl et al., 2018). Previous research indicates that individuals with more significant cognitive resources rely on reasoning and logic. In contrast, those with limited cognitive resources are more prone to relying on emotions and feelings (Shiv, 1999). Meanwhile, according to the Agentic-Communal Model of Power (Rucker et al., 2012) individuals with a high personal sense of power tend to be more inclined toward agentic orientations, which leads individuals to be more self-focused, more concerned with their feelings and more concerned with material possessions that reflect the value of their interests (Rucker et al., 2011). Moreover, people with a high personal sense of power have a stronger utility mentality (Rucker, 2009), and their behavioral intentions tend to be consistent with their values (Magee, 2013). Thus, gift recipients with a high personal sense of power will focus more on the long-term utility or practical value of the gift (Teigen et al., 2005; Galak et al., 2016) rather than the perception of the experience and thus may prefer material gifts that provide them with benefit value; in contrast, individuals with a low personal sense of power tend to develop a communal orientation, which leads individuals to focus more on relationships with others and to consider the feelings of others more when making decisions (Rucker et al., 2011). Meanwhile, communal orientation also leads individuals to pay more attention to warm messages, including honesty, friendliness, tolerance, and sincerity (Dubois, 2016). Based on the Agentic-Communal Model of Power, we identified a matching effect between personal sense of power and the types of gifts received. Specifically, individuals with a high personal sense of power showed a preference for material gifts that align with their interest in utilitarian value and material wealth information (Rucker et al., 2011). Conversely, individuals with a low personal sense of power exhibited a preference for experiential gifts that match their desire for emotional value and warm sentiments. As a result, Individuals with a low personal sense of power may place greater importance on the emotional significance of gifts. They may prefer to give thoughtful and experiential gifts that promote a deeper connection between the giver and receiver. In summary, we derived the following hypotheses:

*H1*: A personal sense of power affects gift preferences.

*H1a*: With a high personal sense of power, individuals will prefer material gifts to experiential gifts.

*H1b*: With a low personal sense of power, individuals will prefer experiential gifts to material gifts.

### Information processing fluency

Based on the above analysis, individuals’ “agentic-communal” orientation in the personal sense of power can result in variations in their attention to information (Dubois, 2016). Individuals with agentic orientation tend to prioritize material wealth and ability information. In contrast, those with a communal orientation tend to emphasize emotional qualities such as trust, patience, friendliness, and sincerity. When forming attitudes, individuals with a high sense of personal power tend to choose the information that is related to material wealth, while those with a low sense of personal power are more likely to rely on information related to passion (Cuddy, 2008; Dubois, 2016). Therefore, during attitude formation, individuals with a high personal sense of power are more likely to select information related to material wealth, while individuals with a low personal sense of power are more inclined to use information associated with warmth and emotions (Cuddy, 2008; Dubois, 2016). Hence, we posit that there is a matching effect between personal sense of power and gift types. Specifically, we suggest that there is a more substantial match between a high personal sense of power and material gifts that offer a high benefit value and convey information about material wealth (Rucker et al., 2011) and a match between a low personal sense of power and experiential gifts with salient emotional attributes (Shiv, 1999). Furthermore, maintaining consistency in matching can enhance the fluency of information processing in individuals (Cesario et al., 2004; Aaker and Lee, 2006). Information processing fluency refers to how easily individuals can process information (Jacoby and Dallas, 1981). When individuals encounter a product that aligns with their behavior habits or values, it will lead to more effortless processing of information related to that product (Winkielman et al., 2003). Therefore, when individuals with a high personal sense of power (or a low personal sense of power) receive material gifts (or experiential gifts) that align with their value orientations, their information processing fluency increases.

The ease of processing information can influence gift recipients’ gift preferences. Firstly, information processing fluency significantly influences people’s perceptions, attitudes, and behaviors. According to Winkielman et al. (2003) information processing fluency has an emotional valence, which tends to trigger positive emotions. This fluency-emotion link can be understood as a “pleasure marker” of processing fluency. Positive emotions experienced by individuals due to information processing fluency can serve as an essential cue for subsequent cognitive evaluations, leading to more favorable evaluations of the processed object, which results in positive emotions, evaluations, and preferences. Furthermore, according to the diagnostic model of attitudes, the ease of extracting information can affect the development of consumer attitudes (Feldman, 1988). A pleasant and smooth experience processing information can contribute to a positive consumer attitude. In other words, information processing fluency can positively impact consumers’ attitudes and behavioral choices. For instance, people tend to consider fluent statements as more credible (Reber and Schwarz, 1999; McGlone and Tofighbakhsh, 2000), and more likable (Bornstein and D'Agostino, 1992), better known (Jacoby and Dallas, 1981). Thus, individuals with a high personal sense of power (or a low personal sense of power) receive a material gift (or experiential gift) consistent with the message of the value, leading to information processing fluency. As a result, they will have a more positive evaluation and preference for the gift.

In summary, we derived the following hypotheses:

*H2*: Information processing fluency mediates the relationship between a personal sense of power and gift preferences.

### Overview of studies

**Figure 1:**
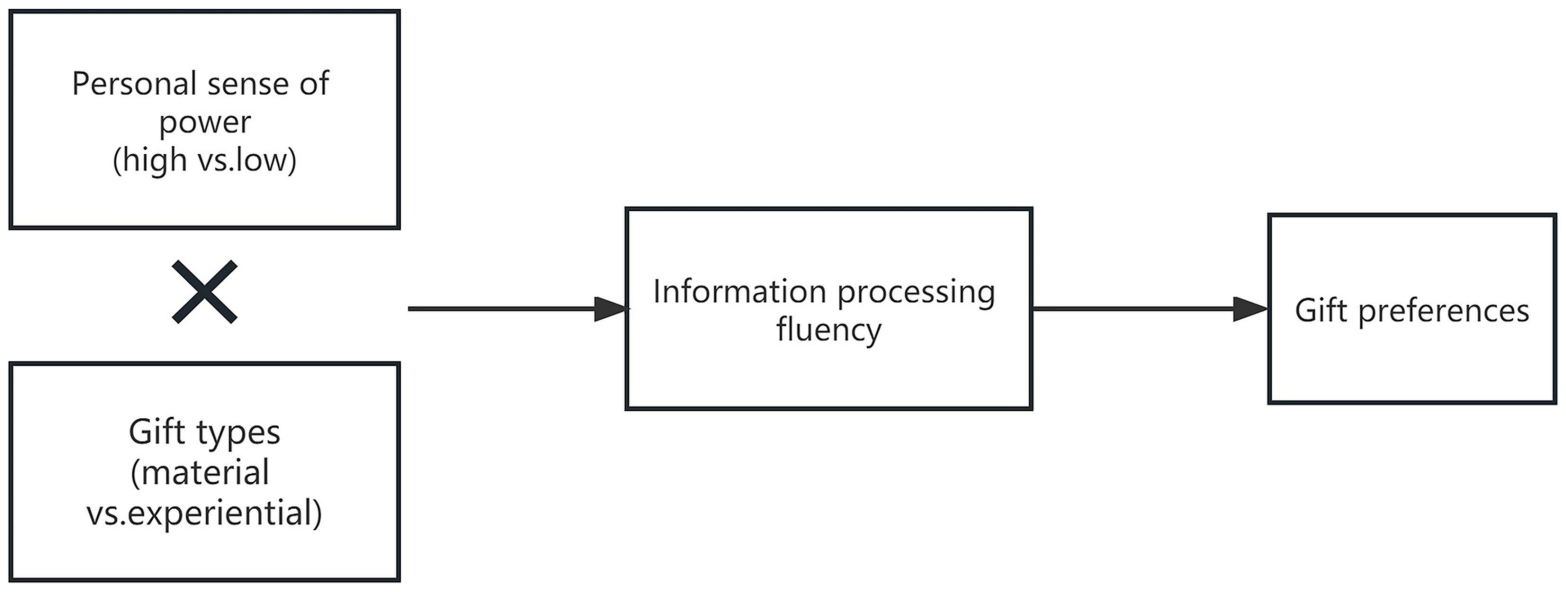
Theoretical model and hypotheses.

Our research model is depicted in Figure 1. We tested our hypotheses in three studies. Study 1 revealed that participants preferred material gifts over experiential gifts when the personal sense of power was high rather than low. In Study 2, we eliminate any explanation of individual personal gift preference to confirm that information processing fluency mediates a personal sense of power and gift preferences. Study 3 involved substituting the experimental materials and altering the initial personal sense of power and further tested the hypotheses that the personal sense of power affects gift preferences.

## Study 1: the effect of personal sense of power on gift preferences

Study 1 was conducted to test H1, which states that a personal sense of power affects gift preference. Specifically, individuals with a high personal sense of power tend to prefer material gifts over experiential gifts. In contrast, individuals with a low personal sense of power preferred experiential gifts over material gifts.

### Pretest study

#### Sample and design

In the pretest experiment, 51 participants were recruited through the “Credamo” platform (similar to Amazon Mechanical Turk) (58% female, age 21–40).

#### Procedure and measures

In the Pretest, we used previous research on experiential and material gifts to guide our selection of experimental materials. We selected 15 gifts, including tickets to tourist attractions, electronic bracelets, tickets to food festivals, watches, buffet coupons, designer perfumes, concert tickets, thermos cups, amusement park play cards, shoulder and neck massages, fitness courses, wallets, shoulder and neck massage coupons, Bluetooth headphones, and spa experience cards, before presenting the experimental materials to participants. We provided an introduction to the concept of experiential and material gifts. We took this step to ensure that all participants comprehensively comprehended the difference between these two types of gifts. This survey referenced the established rating scale for the attributes of gift materials (Goodman et al., 2016). Each participant was asked to rate the “extent to which the gift is more experiential or more material” on a nine-point Likert scale (1 = purely experiential, 9 = purely material). They rated these 15 gifts separately. Finally, we collected participants’ demographic information, such as gender and age.

### Results

Ranking the mean scores of the material attributes for each gift. The top five were material gifts (Wallet, Watch, Thermal mug, Bluetooth headphones, Electronic bracelet), and the bottom five were experiential gifts (Amusement park play cards, Fitness course, Spa card, Concert tickets, Tourist attraction tickets), which served as the stimulus materials for the main experiment.

### Main study

#### Sample and design

A total of One hundred and eighty-three participants were recruited in a university in Southern China (66.1% female, age 18–69, *M*_age_ = 31.33, SD = 8.86), through the “Credamo” platform (similar to Amazon Mechanical Turk), completed this study and received a random monetary payment ranging from $5 to $10. Participants were randomly assigned to 2 (personal sense of power: high vs. low) × 2 (gift type: experiential vs. material), in which personal sense of power is within-group design.

#### Procedure and measures

First, the subjects were asked to imagine that they would receive a holiday gift from a friend and were shown a list of 10 gifts of equal value, five experiential gifts (Amusement park play cards, Fitness course, Spa card, Concert tickets, Tourist attraction tickets) and five material gifts (Wallet, Watch, Thermal mug, Bluetooth headphones, Electronic bracelet). All the above gifts were presented randomly and were not labeled as experiential or material. Subjects were then told that all gifts cost $30 in value. Second, we asked subjects to indicate their preferences by scoring. Third, participants were asked to complete a self-report measure of a personal sense of power adopted by Anderson et al. (2012). Finally, we collected the demographic information of the subjects.

A personal sense of power is defined as an individual’s subjective perception of their ability in social relationships based on their perception of possessing more valuable resources than others. To measure this construct, Anderson et al. (2012) scale was employed for the experiment. The scale contains eight items, such as “I can make people listen to what I have to say.” Subjects assessed the extent to which the statements in each item corresponded to their situation on a 7-point scale (1 = “totally disagree,” 7 = “totally agree,” on a scale from “1” to “7”). Gift preferences are measured through scoring methods. Subjects indicate their preferences for each gift on a 7-point Likert scale (1 = “strongly dislike,” 7 = “strongly like,” on a scale from “1” to “7”) for similar adaptations (see Goodman and Lim, 2018).

### Results

We calculated the average preference scores for experiential and material gifts separately and created a preference score for each type. Then, we conducted a regression analysis with a personal sense of power as the independent variable and gift type preference score as the dependent variable. As predicted, the results indicated that the personal sense of power has a positive impact on the scores of material gifts (*b* = 0.192, *p* = 0.009)；Meanwhile, the personal sense of power has a negative impact on the scores of experiential gifts (*b* = −0.148, *p* = 0.045). The results verified hypothesis 1, which showed that individuals with a high personal sense of power preferred material gifts to experiential gifts. In contrast, individuals with a low personal sense of power preferred experiential gifts to material gifts.

### Discussion

Study 1 demonstrated that individuals with a high personal sense of power preferred material gifts to experiential gifts. In contrast, individuals with a low personal sense of power preferred experiential gifts to material gifts, supporting H1. In addition, Study 1 measures the personal sense of power through the scale of Anderson et al. (2012). Lack of manipulation of the personal sense of power through scenarios. Thus, to address this limitation, hypothesis 1 will be further examined in study 2, which will involve altering the stimuli and manipulating subjects’ personal sense of power in a controlled environment.

## Study 2: information processing fluency mediates the relationship between a personal sense of power and gift preference

The purpose of study 2 was to explore how a personal sense of power affects individuals’ gift preferences and test that information processing fluency serves as a mediator in the correlation between personal sense of power and gift preferences. In the present study, in order to further verify H1, we replaced the product of the experiment, providing a variety of gifts chosen by the subjects individually in Study 1 and a more rigorous test for the connective power of experiential gifts in Study 2, by keeping the gift itself constant and manipulating the framing of the gift solely as either experiential or material, researchers have found that numerous material gifts also encompass experiential elements. Using a luxury perfume as an example, it is not just a tangible item that can be preserved for a long time but also provides an immersive olfactory experience. A book not only serves as a tangible item for people to collect but also provides them with an immersive reading experience. Study 2 leveraged the flexibility between material and experiential gifts and examined whether a material gift, such as a music stereo, could be reframed as a more experiential option by emphasizing the immersive music-listening experience, thus heightening the preference for this type of gift. Second, we employed role-playing to manipulate the participants’ personal sense of power.

### Pretest study

#### Sample and design

In the pretest study, 281 participants were recruited through the “Credamo” platform (similar to Amazon Mechanical Turk) (70.1% female, *M*_age_ = 31.90, SD = 9.86) and offered a gift-wrapped music stereo as a gift to them.

#### Procedure and measures

**Figure 2:**
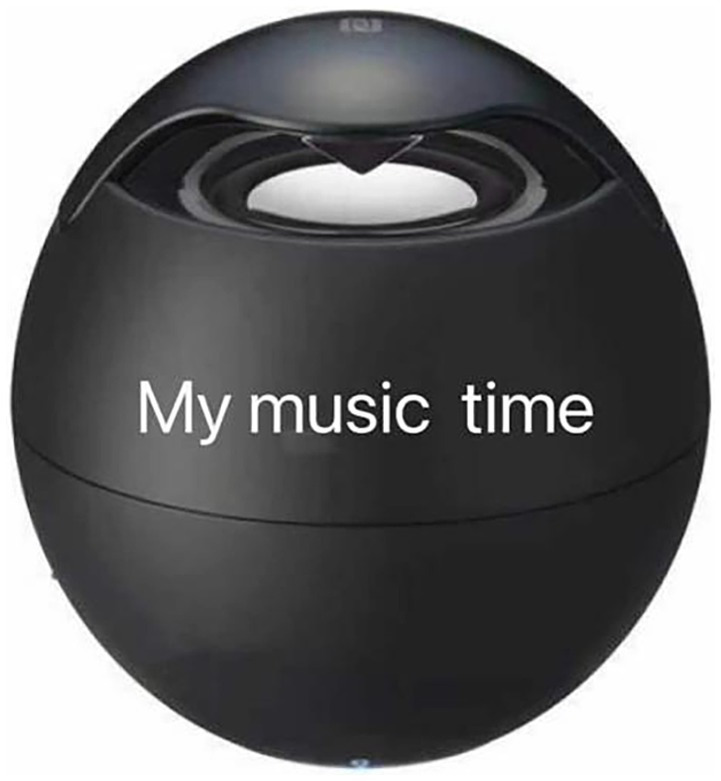
Study 2: the effect of personal sense of power on gift preferences.

**Figure 3:**
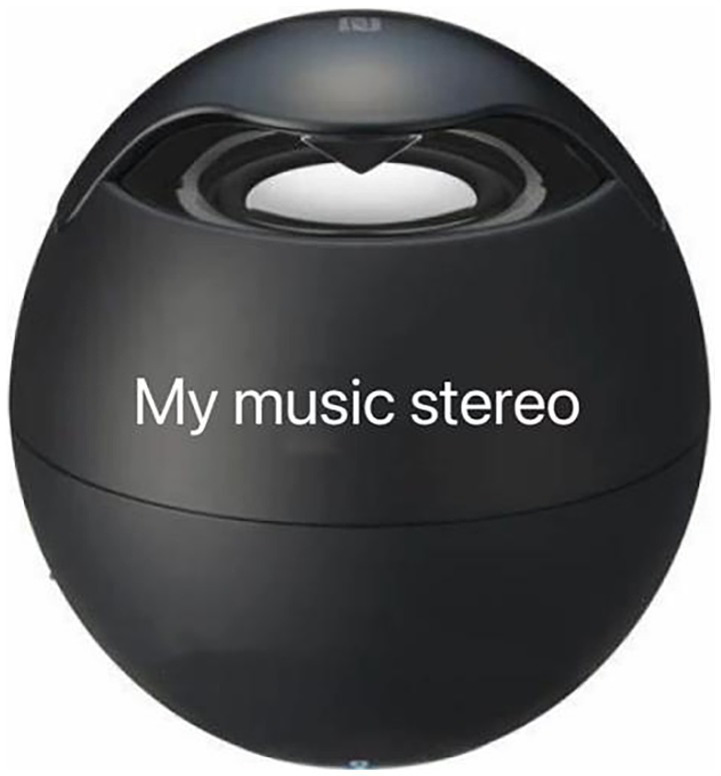
A shoulder and neck massage “compact and portable, one button control, high quality”.

In the Pretest, we used the experimental material with a music stereo that had both experiential and material properties, manipulated its material properties through the slogans “My Music Time” and “My Music Stereo” and the product description, and asked the subjects to rate its material properties separately. Participants were randomly assigned to either give a music stereo that highlighted the experience of listening to music (with the words “my music time” inscribed on it; see Figure 2) or give a music stereo identified as a material possession (with the words “my music stereo” inscribed on it; see Figure 3), the description of the “my music time” highlighted the experiential attribute: play beautiful music and experience your own listening time, the description of the “my music stereo” highlighted the material attribute: a quality mini stereo with a classic player just for you. A pretest between subjects verified the manipulation: Participants were presented with one of the two music stereos and then asked to rate the music stereo on a nine-point scale (1 = “purely experiential,” 9 = “purely material”).

### Results

We made a one-way (ANOVA) test of the material attribute of the gift “My Music Stereo” and the gift “My Music Time” showed that the material attribute scores of the two materials the difference were significant. The material attribute score of “My Music Stereo “(*M* = 6.13, SD = 2.56) was greater than that of “My Music Time” [*M* = 5.05, SD = 2.65, *F*(1, 281) = 11.89, *p* = 0.01]. This result indicates that the gift “My Music Stereo” has a more substantial material attribute and is a material gift. In comparison, the gift “My Music Time” has a more substantial experiential attribute and is an experiential gift.

### Main study

#### Sample and design

One hundred eighty-one subjects (58.6% female, *M*_age_ = 32.70, SD = 10.43) were recruited around a large shopping mall in China Participate in this experiment, through the “Credamo” platform (similar to Amazon Mechanical Turk) participated in this experiment, and each would receive $5 cash as a reward. Subjects were randomly assigned to the high or low personal sense of power group. In order to test our hypothesis, the main experiment used a between-groups factorial experimental design of 2 (personal sense of power: high vs. low) × 2 (gift type: experiential vs. material).

#### Procedure and measures

First, We divided the subjects into two groups with high or low personal sense of power. The subjects in the high (or low) personal sense of power group were shown a picture of a group learning scenario. They were asked to read the following description: “Imagine that you are taking a required course in your major this semester, and the teacher has assigned a group assignment for 70% of the final grade. The group members agree that you (or one of the students) are the most competent, so the teacher appoints you (or him) as the group leader. Next, all other group members will be directed by you (or him) and will complete the group work on time under your (or his) leadership. Also, at the end of the semester, you (or he) will evaluate each group member’s performance and calculate the overall grade. However, the group members will not have the right to evaluate the leader.” After the subjects read the material information, the subjects’ perceived personal sense of power status was measured. Furthermore, subjects’ emotions were measured in order to exclude the effect of a personal sense of power initiation on emotions for this study. Second, subjects were asked to imagine that they would receive a holiday gift from a friend. They were shown the two types of music stereo gifts in the experiment and asked to respond their preferences for experiential gifts and material gifts. Third, we asked subjects to indicate their information processing fluency by scoring. Similarly, to further conceal the purpose of the experiment, we asked them to write down their favorite brand of audio, and after the measurement, we asked them to guess the purpose of the Study. Finally, we collected the subjects’ demographic information, thanked them, and finished the experiment.

Considering that the subjects had previous experience with education, Study 2 drew on Garbinsky et al.’s (2014) method of manipulating the personal sense of power by placing the role play in an educational context and bolded reminders of crucial information. Gift preferences are measured through scoring methods. Subjects indicate their preferences for the music stereo on a 7-point Likert scale (1 = “strongly dislike,” 7 = “strongly like, “on a scale from “1” to “7”) for similar adaptations by Goodman and Lim (2018). Information processing fluency was measured regarding a well-established scale (Kim et al., 2009) by having subjects read the relevant definitions and then proceeding through three question items: “I feel comfortable processing information about the gift I have chosen, I feel comfortable processing information about the gift I have chosen, I feel comfortable processing information about the gift I have chosen. “Subjects assessed the extent to which the statements in each item corresponded to their situation on a 7-point scale (1 = “totally disagree,” 7 = “totally agree,” on a scale from “1” to “7”). And the subjects’ emotions was measured regarding a well-established scale from Watson et al. (1988).

### Results

**Figure 4:**
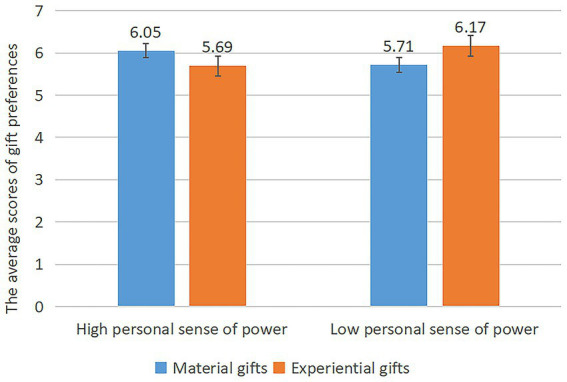
A music stereo that highlighted the experience of listening to music.

First, for the manipulation check, we examined whether the role-play method successfully elicited the subjects’ personal sense of power. The results showed that the personal sense of power scores of the high personal sense of power group (*M* = 6.03, SD = 0.50) and the low personal sense of power group (*M* = 3.51, SD =1.77) was significantly different [*F*(1, 181) =158.15, *p* < 0.000], indicating the success of the personal sense of power manipulation. Next, we conducted a univariate ANOVA on the subjects’ experiential gift and material gift preference scores. The findings demonstrated significant interaction effect of a personal sense of power with gift type [*F*(1, 181) = 9.904, *p* = 0.002]. For those with a high personal sense of power, they showed a higher preference for receiving material gifts than experiential gifts [M material = 6.09, SD = 0.75; M experiential = 5.66, SD = 0.81, *t*(84) = 3.85, *p* = 0.015]; In contrast, for those with a low personal sense of power, they showed a stronger preference for receiving experiential gifts than material gifts (M material = 5.66, SD = 1.17; M experiential = 6.10, SD = 0.84, *t*(97) = 4.59, *p* = 0.039), as shown in Figure 4.

An analysis of variance (ANOVA) was subsequently performed with the information processing fluency score as the dependent variable. The findings indicated no statistically significant main effect of the personal sense of power [*F*(1, 181) = 0.320, *p* = 0.572]. Additionally, the main effect of gift type was found to be non-significant [*F*(1, 181) = 0.177, *p* = 0.674]. However, the interaction effect of the personal sense of power with gift type was significant [*F*(1, 181) = 12.238, *p* = 0.001]. Simple effects analysis found that information processing fluency for experiential gifts was significantly higher than for material gifts for those with a low personal sense of power [*M*_experiential_ = 6.17, SD = 0.68; *M*_material_ = 5.71, SD = 0.82, *t*(97) = 5.01, *p* = 0.004]; for those with a high personal sense of power, information processing fluency was significantly higher for material gifts than for experiential gifts [*M*_material_ = 6.05, SD = 0.53; *M*_experiential_ = 5.69, SD = 1.00, *t*(84) = 2.67, *p* = 0.045]. An ANOVA with mood scores as the dependent variable revealed no significant differences in mood between the individual personal sense of power groups [*F*(1, 181) = 0.647, *p* = 0.422], thus ruling out an effect of mood on the experimental results. Again this effect did not seem to be related to the degree to which the recipients liked the music stereo, as the recipients reported no significant difference in the subjects’ liking of the music stereo either [*F*(1, 181) = 0.003, *p* = 0.96].

Next, In order to investigate the mediating role of information processing fluency, we followed Hayes’ proposed procedure for conducting the mediation analysis (Hayes et al., 2017), using PROCESS model 7 to test the mediating role of information processing fluency in the relationship between personal sense of power and gift preferences. We considered personal sense of power and gift type as categorical variables. A low personal sense of power was coded as 0, while a high personal sense of power was coded as 1. Material gifts were coded as 0, while experiential gifts were coded as 1. The sample size was 5,000 and estimated by the bootstrap method at 95% confidence interval. The results indicated a mediating effect of information processing fluency (LLCI = 0.2345, ULCI = 0.5613, interval not including 0), and the mediating effect size was 0.499. When controlling for the mediating variable information processing fluency, the direct effect of the personal sense of power and gift preference was insignificant (LLCI = −0.2575, ULCI = 0.2641, interval containing 0), indicating that information processing fluency was fully mediating.

### Discussion

Study 2 further supports our hypothesis (Hypothesis 1), which posits that the emphasis on the degree of a personal sense of power influences gift preferences. The study kept all gift characteristics constant except for the level of experiential attributes. The findings suggest that individuals with a high personal sense of power prefer material to experiential gifts. In contrast, individuals with a low personal sense of power will prefer experiential gifts to material gifts. Indeed, even a material gift (a music stereo) could be made more connective by reminding the recipient of the experience it offers (the time spent listening to music). Many gifts have experiential and material elements, and these results demonstrate that gift-givers can enhance the experiential aspects of a gift by emphasizing the experiential enjoyment it provides. Additionally, Study 2 confirmed our mediating hypothesis (Hypothesis 2), which proposes that information processing fluency mediates the relationship between personal sense of power and gift preference.

## Study 3: manipulated power and birthday gift-giving context

The preceding two studies offered empirical support for Hypothesis 1, showing that a personal sense of power affects individuals’ gift preferences. Moreover, Study 2 verified our mediating hypothesis (Hypothesis 2). In the following Study 3, we will replace the experimental product and replace the way to initiate the personal sense of power and further verify the main effect. To overcome the limitations of the holiday gift-giving context used in Study 1, Study 3 set up the context as a birthday gift-giving context, which was closer to daily life, and replaced the experimental product to improve the generalizability of the experimental findings.

### Method

#### Sample and design

Two hundred and fifty-one postgraduate students in China were recruited through the “Credamo” platform (68.9% female, *M*_age_ = 30.57, SD = 7.65) participated in this experiment, and each would receive $5–10 cash as a reward. To test our hypothesis, we used a between-groups factorial experimental design of 2 (personal sense of power: high vs. low) × 2 (gift type: material vs. experiential). Subjects were randomly allocated to either the high or low personal sense of power group.

#### Procedure and measures

**Figure 5:**
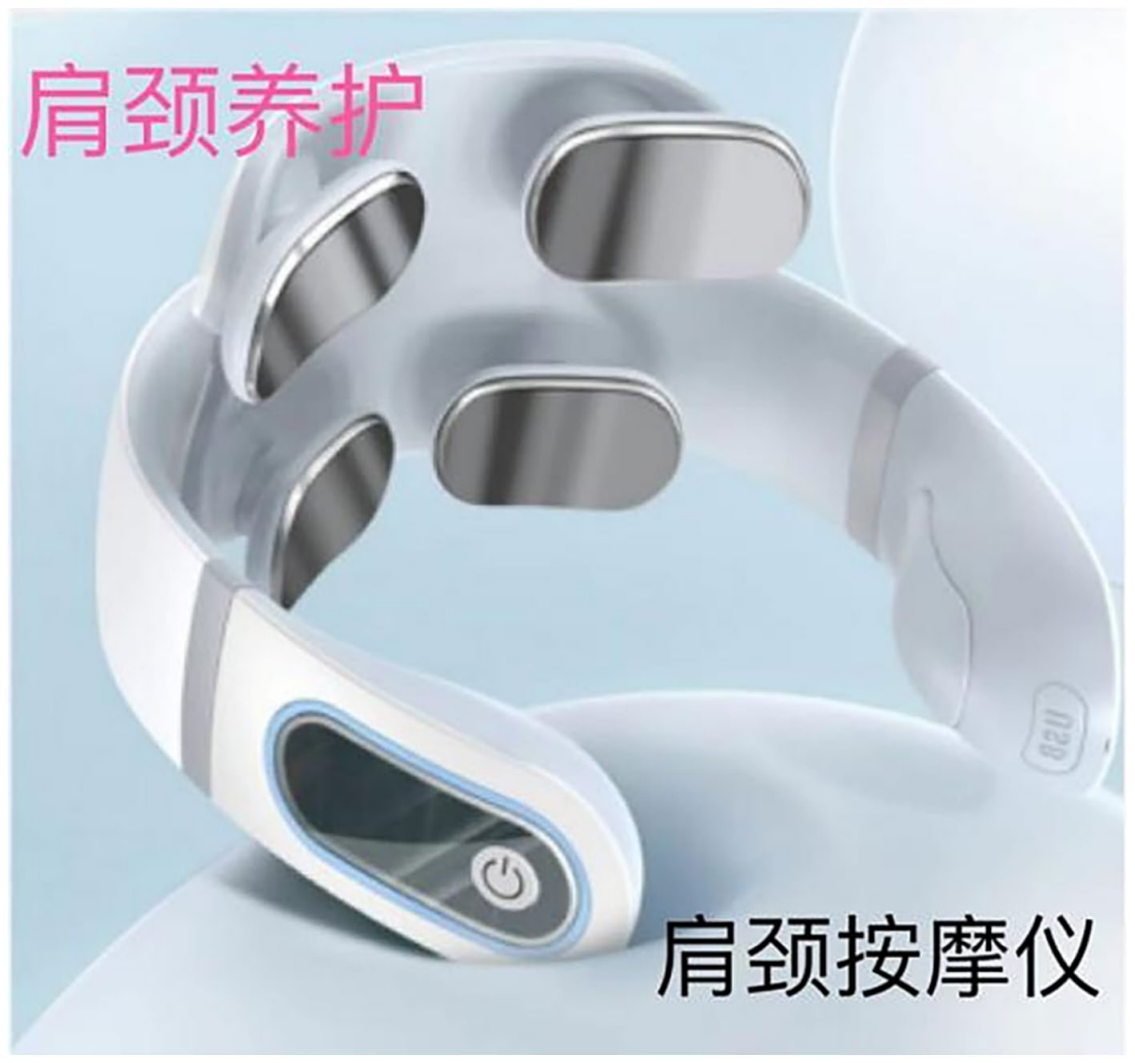
A shoulder and neck massage coupon ”deep pressure, cervical spine physiotherapy, soothing body, and mind”.

**Figure 6:**
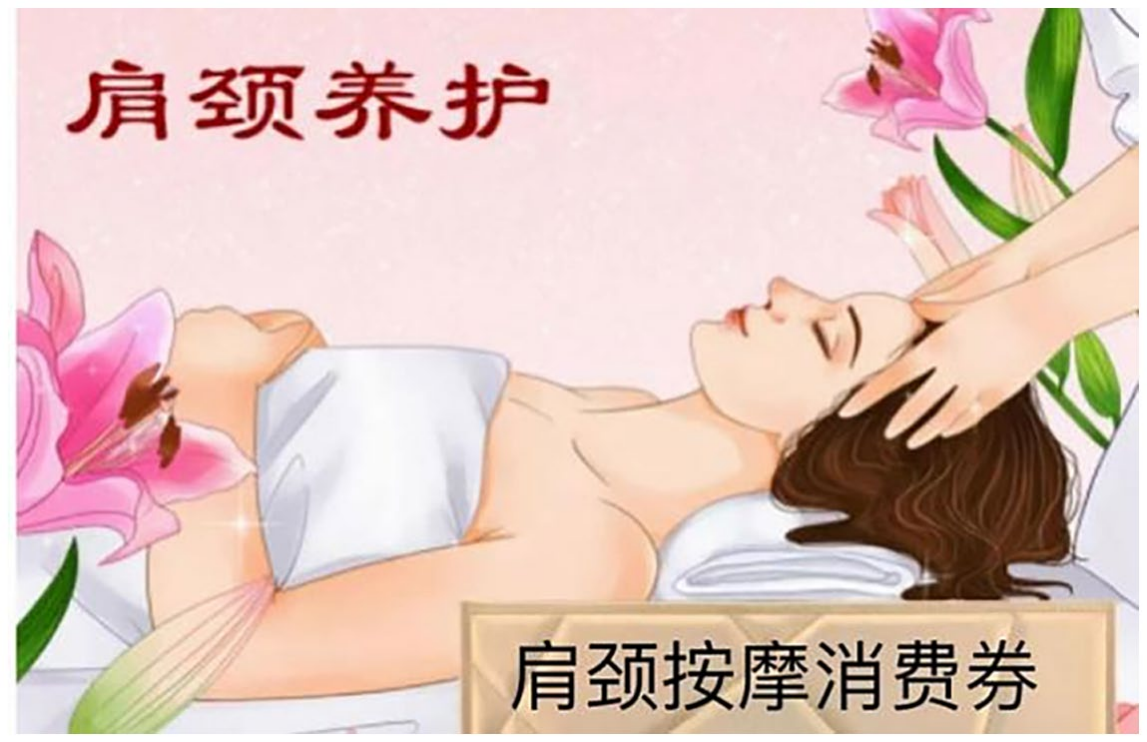
Study 3: the effect of personal sense of power on gift preferences.

First, we divided the subjects into two groups with high or low personal sense of power. For the initiation of a high (or low) personal sense of power, “recall a scene or example where you (or another person) were very powerful over another person (or you). Power here refers to situations where you (or others) can control or influence others (or you) to get what they (or you) want or where you (or others) judge others (or you). Please explain in as much detail as possible what happened, how you felt.” Moreover, to show the subject pictures of scenarios in which others (or you) are compelling. Then we measured the subjects’ perceived personal sense of power status. After the personal sense of power manipulation was completed, subjects were given a decision situation for gift selection. After first having the subjects read the relevant materials on the definitions of experiential gifts and material gifts. We provided two gifts to the subjects and introduced them. Gift A was a shoulder and neck massage labeled “compact and portable, one button control, high quality” (see Figure 5). At the same time, Gift B was a shoulder and neck massage coupon labeled “deep pressure, cervical spine physiotherapy, soothing body, and mind” (see Figure 6). To ensure that the difference between the two gifts in terms of function is as slight as possible, the introduction of the two groups also tried to maintain a similar number of words to avoid influencing the subjects’ choice as much as possible. Second, we asked subjects to indicate the attributes of gift materials by scoring. Third, we asked the subjects to chose between the two gifts offered, indicating a preference for which gift they would like to receive as an upcoming birthday gift. Finally, subjects were also asked to report whether they had ever received a gift, and those who had yet to experience receiving a gift were treated as invalid. The manipulation check method was the same as in Study 2. Upon the conclusion of the experiment, subjects were asked to guess the experiment’s purpose and write demographic information data.

Study 3 initiated the subjects’ personal sense of power by recalling a particular incident method (Galinsky et al., 2003). Participants rated the personal sense of power by setting three questions, with the central question being “I felt full of a personal sense of power in the above-recalled scenario.” Subjects assessed the extent to which the statements in each item corresponded to their situation on a 7-point scale (1 = “strongly disagree,” 7 = “strongly agree”) adapted from Faul et al. (2009). And each participate scored the material attributes of Gift A and Gift B (1 = “purely experiential,” 9 = “purely material”), as a way to detect differences in the experiential attributes of the experimental materials adopted by Goodman and Lim (2018). As for the measurement of gift preferences, participants were presented with two gifts and asked to make a selection between them. The proportion of choices for each gift was recorded as the outcome of the experiment.

### Results

A manipulation check was first conducted. We examined whether the recalling a particular incident method was successful in eliciting the subjects’ personal sense of power. The findings showed that the high personal sense of power group (*M* = 5.90, SD = 1.06) and the low personal sense of power group (*M* = 2.67, SD = 1.64) were significantly different [*F*(1, 251) = 336.34, *p* < 0.000], indicating the success manipulation of the personal sense of power. Gift type manipulation test: material attribute score of Gift A shoulder and neck massage instrument (*M* = 7.70, SD = 1.72), Gift B shoulder and neck massage consumer coupon material attribute score (*M* = 2.34, SD = 1.87). The difference in material attribute scores between the two gifts was found to be statistically significant [*F*(1, 251) = 1115.59, *p* < 0.000], which proved the success of gift type manipulation.

**Figure 7:**
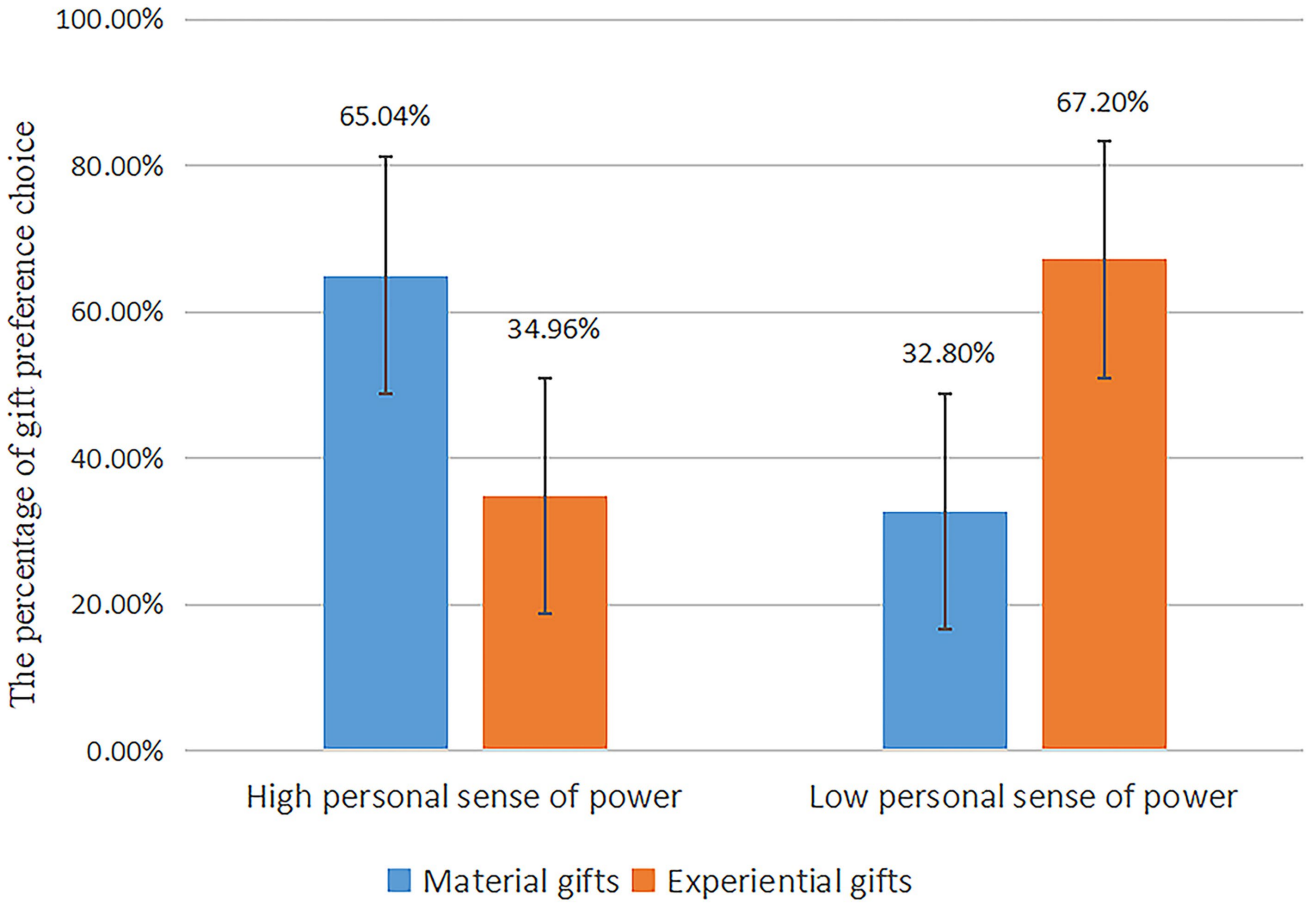
A music stereo identified as a material possession.

We performed a chi-square test, using the personal sense of power as the independent variable and gift type selection as the dependent variable. The results showed that the proportion of subjects choosing experiential gifts (67.2%) was more significant than the proportion choosing material gifts (32.8%) in the low personal sense of power group [Gift A = 32.8% vs. Gift B = 67.2%; χ^2^(1) = 15.125, *p* < 0.000]. The proportion of subjects choosing material gifts (65.04%) was more significant than the proportion choosing experiential gifts (34.96%) in the high personal sense of power group [Gift A = 65.04% vs. Gift B = 34.96%; χ^2^(1) = 11.13, *p* = 0.001]. The difference was statistically significant [χ^2^(1) = 26.08, *p* < 0.000], again verifying the main effect, as shown in Figure 7.

Finally, we conducted a variable control test: Although this study focused on the effect of a personal sense of power on gift preference, studies have also suggested the effect of price on gift preference, which is used as a control variable in this study. Although in the experiment we informed subjects that the gifts presented were identical in price, in order to better test whether the price factor influenced the subjects’ decision when deciding after the subjects made their choice, we asked them to report whether they chose the gift because it appeared to be more expensive. The results showed that 15.8% of the subjects who chose material gifts made their choice because they seemed expensive, and 12.8% of the subjects who chose experiential gifts made their choice because they seemed expensive, and the effect of expensive (yes vs. no) on the experimental results was not significant [Gift A = 15.8% vs. Gift B = 12.8%; χ^2^(1) = 0.017, *p* = 0.897].

### Discussion

Study 3 provided additional support for our main hypothesis using a highly conservative. Which posits that the emphasis on the degree of a personal sense of power influences gift preferences. The results suggest that individuals with a high personal sense of power prefer material to experiential gifts as birthday gifts. Study 3 also showed that the price factor of the gift had no effect on the study results.

## General discussion

There have been numerous studies conducted on individual gift preferences. However, they tend to primarily focus on factors such as social distance, gender, level of explanation, age, income, interpersonal orientation, and cultural background (Parsons, 2002; Rucker et al., 2012; Puente Díaz and Cavazos, 2021). Less on a personal sense of power. Our studies address existing research gaps by investigating how the personal sense of power affects gift preferences. Specifically, we propose and provide evidence for the following mechanism: Experiential gifts enhance the information processing fluency for individuals with a low personal sense of power, leading to a preference for experiential gifts.

For individuals with a high personal sense of power, material gifts (or experiential gifts) are associated with greater information processing fluency and a preference for material gifts. Through three experiments, this paper investigates the impact of a personal sense of power on gift preferences. For Study 1, ten gifts (five material and five experiential gifts) were selected as experimental materials to validate that variances in a personal sense of power influence individuals’ gift preferences (hypothesis 1). In Study 2, we employed a singular item (a music stereo, serving as both a material and experiential gift) as the gift-receiving context. Study 2 used musical stereo as the experimental material to keep the gift constant while changing only the experiential and material frames of the gift. This approach aimed to eliminate interference from factors such as participants’ preexisting preferences for different gifts. The goal was to test further hypothesis 1, which suggests that a personal sense of power (high vs. low) affects their gift preferences (material gifts vs. experiential gifts). In addition, this study confirmed hypothesis 2, which posits the mediating effect of information processing fluency in the relationship between personal sense of power and gift preferences. Furthermore, Study 3 utilized a birthday gift-giving scenario, which closely resembles everyday situations, In order to ensure comprehensive representation of different types of gifts, Experiment 3 substituted both tangible and intangible gifts as experimental materials to enhance the generalizability of the experimental results.

### Contribution and implication

First, while there has been some research conducted in the domains of psychology and marketing about a personal sense of power (Anderson and Berdahl, 2002; Aaker and Lee, 2006; Sijbom and Parker, 2020; Zheng et al., 2023), information processing fluency (Freddi et al., 2014; Aubrey, 2022; Cao et al., 2022), and individual gift preferences (Teigen et al., 2005; Aubrey, 2022; Cao et al., 2022). However, fewer scholars have explored the direct effects of a personal sense of power (high vs. low) on individual gift preferences (material gifts vs. experiential gifts). The present study offers an empirical test of the issues mentioned earlier, making an innovative theoretical contribution. The following details outline the study’s approach. First, this paper proposes new influences on individual gift preferences and explores the underlying mechanisms. The existing literature on individual gift preferences focuses on social distance (Goodman and Lim, 2018), gender (Parsons, 2002; Pollmann and van Beest, 2013), age and income (Parsons, 2002; Dong et al., 2023), interpersonal orientation (De Hooge, 2017; Liao et al., 2023), and culture (Wang, 2007; Aung et al., 2017). While many factors influence gift preferences in interpersonal interactions, a scarcity of studies that have specifically examined the influence of a personal sense of power. This study addresses this gap by exploring how a personal sense of power relates to gift preferences for material vs. experiential gifts. Our findings contribute to the existing research on individual gift preferences.

Second, this paper expands the investigation of a personal sense of power by exploring its relevance to individual gift preferences. The current findings emphasize a personal sense of power as an actual psychological state for individuals, mainly studying its influence on consumption behavior (Rucker et al., 2012)and preference studies for different product choices (Rucker and Galinsky, 2008). This paper investigates how a personal sense of power affects gift preferences. Previous studies on the link between a personal sense of power and gift preferences need to be more conclusive. For instance, while some research suggests that a high personal sense of power leads individuals to prioritize the utility of a gift (Rucker, 2009), this may also cause them to focus less on the experiential aspects of the gift and more on its perceived value. These findings suggest that a personal sense of power may play a role in shaping gift preferences. This paper examining the preference of gifts and showing that individuals with a high personal sense of power prefer material gifts. In contrast, those with a low personal sense of power prefer experiential gifts. These findings offer insights for future research on a personal sense of power and gift-giving preferences.

Third, the research in this paper guides gift-givers and gives insights into individuals’ gift preferences. We assess satisfaction with receiving different types of gifts from the perspective of the gift recipient by conducting several experiments involving various real-life gift exchanges and measures of individual gift preferences (material vs. experiential gifts). Our research consistently shows that experiential gifts prefer individuals with a low personal sense of power over material gifts (Study 1). This effect was confirmed when the same gifts were categorized as relatively more experiential (Study 2). Our findings suggest that giving a gift that matches individuals’ personal sense of power leads to greater satisfaction.

Finally, our research on personal sense of power and gift preferences provides practical insights for gift sales. Marketing professionals can tailor gift selections based on personal sense of power, thereby better meeting customers’ needs. Additionally, for individuals and organizations, choosing appropriate gifts for specific occasions holds significant importance. Understanding the relationship between personal sense of power and gift preferences can help people make wiser choices when giving and receiving gifts, avoiding potential misunderstandings and conflicts.

### Limitations and future research

This study has some limitations. In this study, we employed several methods to stimulate participants’ personal sense of power. However, it should be noted that a personal sense of power can manifest in different forms, and exploring how the personal sense of power feeling influences an individual’s gift preferences is a valuable area for future investigation. Prior research has categorized personal sense of power feelings based on their sources and characteristics. For instance, some scholars have distinguished between rewarding and punitive forms of a personal sense of power. Therefore, it would be worthwhile to consider these distinctions when examining the relationship between a personal sense of power and gift preferences (Hinkin and Schriesheim, 1994), While other scholars have categorized the personal sense of power based on whether individuals perceive it as an opportunity or a responsibility (Scholl et al., 2015). In this study, we provided a brief comparison between individuals with a high and low personal sense of power. However, we did not conduct a thorough analysis of how different types of personal sense of power may influence gift preferences. Therefore, in future studies, it needs to classify personal sense of power based on its formation basis and explore whether the various types of a personal sense of power have varying effects on individuals’ gift preferences.

Second, this paper solely focuses on the gift recipient’s personal sense of power and does not consider the impact of the gift giver’s personal sense of power on the recipient’s gift preference. Specifically, whether individuals with a low personal sense of power still prefer giving experiential gifts to others and whether individuals with a high personal sense of power prefer giving material gifts were examined. Consequently, further investigation is needed to explore the effects of varying personal sense of power between gift recipients and gift givers on individual gift preferences. Therefore, future studies will delve into this area to gain deeper insights.

Finally, this paper primarily employs experimental methods to conduct research. In the experimental design, the limited number of items for measurement has resulted in the need for increased reliability. Field experiments can be carried out to enhance the external validity of future studies. In this paper, we manipulated the subjects’ personal sense of power through reading materials and scene picture stimuli. Although we successfully achieved the manipulation effect while controlling for other factors related to gift preferences, we did not observe actual consumption behavior based on gift reception. Therefore, future research can observe individuals’ gift preferences in a realistic gift-receiving situation, considering different culturally-derived personal senses of power. Conducting field experiments based on real-life situations will result in more realistic and practical findings, enhancing the study’s external validity.

## Data availability statement

The raw data supporting the conclusions of this article will be made available by the authors, without undue reservation.

## Author contributions

SL, XH, and XY contributed to conception and design of the study. SL organized the database. XY performed the statistical analysis and wrote the first draft of the manuscript. ML, YZ, ZL, and PX wrote sections of the manuscript. All authors contributed to the article and approved the submitted version.

## Funding

National Natural Science Foundation of China (Grant No. 72162002 and 72232003); the Science Foundation of Ministry of Education of China (Grant No. 18YJC630083); Guangxi Higher education undergraduate teaching reform project (Grant No. 2021JGA120); the Natural Science Foundation of Guangdong Province of China (Grant No. 2018A030310343); Key Research Base of Humanities and Social Sciences in Guangxi Universities and Guangxi Development Strategy Institute (Grant No. 2023GDSIZD01); the Interdisciplinary Scientific Research Foundation of Applied Economics of GuangXi University (Grant No. 2023JJJXB14); Henan Province Philosophy and Social Science Planning Project (Grant No. 2021BJJ110); Guangdong Province Philosophy and Social Science Planning Project (Grant No. GD20CGL47), and National Social Science Fund Projects (Grant No. 21XMZ081).

## Conflict of interest

The authors declare that the research was conducted in the absence of any commercial or financial relationships that could be construed as a potential conflict of interest.

## Publisher’s note

All claims expressed in this article are solely those of the authors and do not necessarily represent those of their affiliated organizations, or those of the publisher, the editors and the reviewers. Any product that may be evaluated in this article, or claim that may be made by its manufacturer, is not guaranteed or endorsed by the publisher.
