# Supplementary material for: Does having more power make people more materialistic? The role of personal sense of power for gift preferences
Source: Front Psychol. 2023 Aug 25;14:1235527. doi: 10.3389/fpsyg.2023.1235527 (PMC10485253; doi:10.3389/fpsyg.2023.1235527)
Supplement: Supplementary file 1 [file Table_1.DOCX]

| Study | Sample | Design | Manipulation | Measure | Main Purposes |
| --- | --- | --- | --- | --- | --- |
| 1 | 183 participants | 2(personal sense of power： high vs. low) × 2(gift type: experiential vs. material) | 1. Imagine receive a holiday gift from a friend 2. Five experiential gifts and five material gifts 3. Personal sense of power measure | Gift preference scale | Test that personal sense of power affects gift preference |
| 2 | 181  subjects | 2 ( personal sense of power: high vs. low) × 2( gift type: material vs. experiential ) | 1. Manipulate a personal sense of power through role-play 2. Only a single gift is provided，and manipulating the material and experiential of the gift itself | Gift preference scale | 1. Test that information processing fluency serves as a mediator 2. Eliminate the interference of choosing too many personal gift preferences 3. Supplement the manipulation scenario of personal sense of power |
| 3 | 251 participants | 2 ( personal sense of power: high vs. low) × 2( gift type: material vs. experiential ) | 1. Initiated personal sense of power by recalling a particular incident 2. Imagine receive a birthday gift from a friend 3. Make a selection between two gifts | Gift choice | 1. Test that personal sense of power affects gift preference 2. Study 2 provided only a tangible gift, and Study 3 provided both tangible and intangible gifts 3. Supplement the manipulation scenario of personal sense of power |

Appendix A

Table 1.Overview of studies
