# Supplementary material for: Does having more power make people more materialistic? The role of personal sense of power for gift preferences
Source: Front Psychol. 2023 Aug 25;14:1235527. doi: 10.3389/fpsyg.2023.1235527 (PMC10485253; doi:10.3389/fpsyg.2023.1235527)
Supplement: Supplementary file 2 [file Table_2.DOCX]

| Personal Sense of Power Scale | | | | | | | | |
| --- | --- | --- | --- | --- | --- | --- | --- | --- |
| Number | Question items | Totally disagree | Compare disagree | A little disagree | Neutrality | A little  agree | Compare agree | Totally agree |
| 1 | I can let others listen to what I say | 1 | 2 | 3 | 4 | 5 | 6 | 7 |
| 2 | My words didn't do much work | 1 | 2 | 3 | 4 | 5 | 6 | 7 |
| 3 | I can let other people do what I want | 1 | 2 | 3 | 4 | 5 | 6 | 7 |
| 4 | Even if I say my opinion has little difference | 1 | 2 | 3 | 4 | 5 | 6 | 7 |
| 5 | I think I have a lot of influence | 1 | 2 | 3 | 4 | 5 | 6 | 7 |
| 6 | My thoughts and opinions are often overlooked | 1 | 2 | 3 | 4 | 5 | 6 | 7 |
| 7 | No matter how hard I try, it is still difficult for me to follow my own wishes | 1 | 2 | 3 | 4 | 5 | 6 | 7 |
| 8 | I'm very appealing | 1 | 2 | 3 | 4 | 5 | 6 | 7 |

Appendix B

Table 2.Personal Sense of Power Scale
