# Supplementary material for: Does having more power make people more materialistic? The role of personal sense of power for gift preferences
Source: Front Psychol. 2023 Aug 25;14:1235527. doi: 10.3389/fpsyg.2023.1235527 (PMC10485253; doi:10.3389/fpsyg.2023.1235527)
Supplement: Supplementary file 3 [file Table_3.DOCX]

Appendix C

Table 3.Information Processing Fluency Scale

| Information Processing Fluency Scale | | | | | | | | |
| --- | --- | --- | --- | --- | --- | --- | --- | --- |
| Number | Question items | Totally disagree | Compare disagree | A little disagree | Neutrality | A little  agree | Compare agree | Totally agree |
| 1 | I think the information of this gift works very smoothly. | 1 | 2 | 3 | 4 | 5 | 6 | 7 |
| 2 | I think the information of this gift is very easy to process. | 1 | 2 | 3 | 4 | 5 | 6 | 7 |
| 3 | I find the information of this gift very comfortable in processing. | 1 | 2 | 3 | 4 | 5 | 6 | 7 |

| Gift Preferences Scale | | | | | | | | |
| --- | --- | --- | --- | --- | --- | --- | --- | --- |
| Number | Question items | Totally disagree | Compare disagree | A little disagree | Neutrality | A little  agree | Compare agree | Totally agree |
| 1 | I prefer to receive the music sound in the picture (My music stereo) | 1 | 2 | 3 | 4 | 5 | 6 | 7 |

Table 4.Gift Preferences Scale
